# Simultaneous LC/MS Analysis of Carotenoids and Fat-Soluble Vitamins in Costa Rican Avocados (*Persea americana* Mill.)

**DOI:** 10.3390/molecules24244517

**Published:** 2019-12-10

**Authors:** Carolina Cortés-Herrera, Andrea Chacón, Graciela Artavia, Fabio Granados-Chinchilla

**Affiliations:** 1 Centro Nacional de Ciencia y Tecnología de Alimentos, Universidad de Costa Rica, Ciudad Universitaria Rodrigo Facio, 11501-2060 San José, Costa Rica; andrea28.chacon@gmail.com (A.C.); graciela.artavia@ucr.ac.cr (G.A.); 2 Centro de Investigación en Nutrición Animal (CINA), Universidad de Costa Rica, Ciudad Universitaria Rodrigo Facio, 11501-2060 San José, Costa Rica; fabio.granados@ucr.ac.cr

**Keywords:** avocado, LC/MS, fat-soluble vitamins, carotenoids

## Abstract

Avocado (a fruit that represents a billion-dollar industry) has become a relevant crop in global trade. The benefits of eating avocados have also been thoroughly described as they contain important nutrients needed to ensure biological functions. For example, avocados contain considerable amounts of vitamins and other phytonutrients, such as carotenoids (e.g., β-carotene), which are fat-soluble. Hence, there is a need to assess accurately these types of compounds. Herein we describe a method that chromatographically separates commercial standard solutions containing both fat-soluble vitamins (vitamin A acetate and palmitate, Vitamin D_2_ and D_3_, vitamin K_1_, α-, δ-, and γ-vitamin E isomers) and carotenoids (β-cryptoxanthin, zeaxanthin, lutein, β-carotene, and lycopene) effectively (i.e., analytical recoveries ranging from 80.43% to 117.02%, for vitamins, and from 43.80% to 108.63%). We optimized saponification conditions and settled at 80 °C using 1 mmol KOH L^−1^ ethanol during 1 h. We used a non-aqueous gradient that included methanol and methyl *tert*-butyl ether (starting at an 80:20 ratio) and a C_30_ chromatographic column to achieve analyte separation (in less than 40 min) and applied this method to avocado, a fruit that characteristically contains both types of compounds. We obtained a method with good linearity at the mid to low range of the mg L^−1^ (determination coefficients 0.9006–0.9964). To determine both types of compounds in avocado, we developed and validated for the simultaneous analysis of carotenoids and fat-soluble vitamins based on liquid chromatography and single quadrupole mass detection (LC/MS). From actual avocado samples, we found relevant concentrations for cholecalciferol (ranging from 103.5 to 119.5), δ-tocopherol (ranging from 6.16 to 42.48), and lutein (ranging from 6.41 to 15.13 mg/100 g dry weight basis). Simmonds cultivar demonstrated the higher values for all analytes (ranging from 0.03 (zeaxanthin) to 119.5 (cholecalciferol) mg/100 g dry weight basis).

## 1. Introduction

Avocado represents a billion-dollar industry, the projection of the apparent *per capita* consumption of avocado sets the top six world importers of avocado to be US, Netherlands, France, United Kingdom, Spain, and Canada with 3.64, 1.62, 2.10, 2.21, 2.30, and 2.55 kg in a given year, respectively [1]. Avocado exports have made some countries like Mexico, Dominican Republic, Peru, Chile, Colombia, and Costa Rica increase their cultivated area [1].

The topmost exporter countries have directed their offer to destinations such as the US, Europe, and Asia, especially China [2], where import growth is in the order of 250%, from 154 tons in 2012, to 25,000 tons in 2016 [3,4]. For example, Costa Rican avocado harvested area was estimated at 1 888 ha in 2014 and increased to 3092 ha in 2017, which represents 845 tons of Costa Rican avocado (or 448900 USD) [5].

As a complex matrix, performing chemical analysis on the avocado flesh presents an additional difficulty. The ripe avocado fruit has a firm, oily, and yellow to light green colored mesocarp that contains both fat-soluble vitamins and carotenoids associated with other lipids [6]. Usually, both types of analysis have to be performed separately using different chromatographic conditions altogether, which represents an additional expense, both economic and in terms of labor. Analyzing both types of fat-soluble compounds is relevant, especially in fruits such as avocado in which there is evidence that carotenoid absorption might be improved by the addition of avocado and avocado oil [7,8,9].

Interestingly, the Association of Official Analytical Chemists (AOAC) Official Methods of Analysis (OMA^SM^) does not have any method established for neither fat-soluble vitamins nor carotenoids in fruits. Several papers have already annotated the relevance of including avocado in the diet as it has been related to health benefits [10,11,12,13,14,15,16,17,18,19]. Previously, another report analyzed carotenoid content in avocado using spectrophotometry [20]. A later report has used a similar approach to measure total carotenoid content [21]. On another hand, a research group has analyzed avocado pigments in oil [22] and tissue [22,23] using HPLC coupled with a photodiode array detector using a triphasic organic solvent gradient. Carotenoids in avocado seed have also been described [24,25].

The application of chromatography and mass spectrometry to carotenoid analysis in fruits is not new [23,26,27]. However, few papers have been dedicated solely to the study of both fat-soluble and carotenoid content in avocado fruits. For example, research assayed both types of compounds (i.e., carotenoids and tocopherol) using two independent chromatographic techniques [28].

Among the papers dedicated to assessing specifically carotenoids or vitamins in avocado, the most relevant include advantages such as that most researchers use acetone (a versatile and low boiling point solvent) during primary extraction [22,23,28,29] and ethyl ether or hexane after saponification [22,23,29]. For saponification at room temperature, the use of 2,6-di-*tert*-butyl-4-methylphenol, and nitrogen flushing seems to be a norm [28,29], thus protecting the target analytes. Diversity of carotenoids studies is ample (including epoxides [28], isomers of carotene [20], chlorophylls [22], phytoene [23]). Mobile phases are usually simple and environmentally friendly [22,23,29], which include methanol, water, methyl *tert*-butyl ether, and ethyl acetate. Yano and coworkers were able to apply their method to 75 and 15 different fresh and processed fruits, respectively [23]. Solid-phase extraction has been used to reduce interferences [22]. Mass spectrometry has been used to recognize unidentified compounds [20,28].

Disadvantages of these methods include the presence of water in the mobile phase (which increases mobile phase polarity), several approaches exhibit some issues with chromatographic resolution for some of the signals [28,29] and in some cases saponification time [29], base concentration [29], and chromatographic run [23] are excessive or the identification of compounds is based on light absorption [20,22,29]. Finally, vitamin analysis of the fruit is scarce at best [11,28].

Lastly, very little information has been gathered regarding varieties of Costa Rican avocados, proximate analysis, mineral content, and some vitamins have been explored [30]. Some papers have also focused on standing out differences between avocado varieties [21,28], including varieties of Guatemalan race (e.g., Hass [11] and Nabal [20]), as avocadoes originated from New Zealand [22], California [28], and Mexico [29] and those commercially available from Israel [20] and Japan [23].

Herein, we report a liquid chromatography and single quadrupole mass detection (LC/MS) based method using a C_30_ column and methanol and methyl *tert*-butyl ether to assay and quantitatively separate both fat-soluble vitamins (vitamin A acetate and palmitate, Vitamin D_2_ and D_3_, vitamin K_1_, α-, δ-, and γ-vitamin E isomers) and carotenoids (β-cryptoxanthin, zeaxanthin, lutein, β-carotene, and lycopene) simultaneously in avocado fruit.

## 2. Results and Discussion

### 2.1. Stationary Phase Selection and Green Chemistry

Where other alkyl modified stationary phases failed (Table 1), the C_30_ allowed an excellent separation of fat-soluble compounds even between structurally related molecules using a MeOH/*tert*-butyl methyl ether (MTBE)-based mobile phase (Table 2). The solvent selection not only ensured good compound solubility and chromatographic separation, but it also helped improve column life span as no water was involved and the column could be safely stored under 80% MeOH.

Some methods have selected chlorinated solvents as an effective way to extract [28] or separate carotenoids [31]. However, we chose MTBE as a greener alternative to chlorinated solvents [32]. Additionally, our chromatographic separation was mostly based on MeOH. The high degree of shape recognition of the C_30_ was validated early [33] and was, once again, here, demonstrated. It is recommended for analysis of retinoid as well as carotenoid molecules [34]. However, herein, we exploited the versatility of the C_30_ column further. Furthermore, as avocado lacks the presence of lycopene [20,28], this compound when introduced into the separation (especially in its deuterated form), can be used as an internal standard (IS). Though lycopene is considered a relative liable carotenoid, it has been used successfully as an IS [35]. Other more stable molecules have been used as well and could be considered (e.g., canthaxanthin [35], sudan I [36]; 8′-apo-8′-β-carotenal [37], echinenone [38]).

### 2.2. Singular Ion Monitoring Parameter Selection

As expected, using reverse phase chromatography, fat-soluble vitamins eluted during the first 8 min (except for retinyl palmitate, an esterified compound with an extra C_15_ alkyl chain) (Table 2). Both retinoids assessed were prone to retain monovalent cations such as K^+^ ([M + K]^+^). Retinyl acetate and retinyl palmitate share a similar fragmentation pattern, which includes ion 269 *m*/*z* as a base peak (Table 2). The retinoid alkyl chain did not seem to affect ionization voltage, indicating that the C_20_H_29_OH base was governing their behavior. Ion 296 *m*/*z* found for retinoids corresponded to the protonation and elimination of water and acetate during positive ion electrospray [34].

All tocopherol isomers, as well as zeaxanthin, β-cryptoxanthin, β-carotene, and lycopene (the heavier analogs), exhibited deprotonated species as molecular ions ([M+]). Electrospray ionization (ESI^+^) ions are usually preformed in solution by acid/base reactions (i.e., [M + nH]^n+^), some ions are probably formed by a field desorption mechanism at the surface. Hence, the production of abundant cations, with little fragmentation [34]. Meanwhile, phylloquinone, the acidic carotenoid astaxanthin [34], and lutein all ionized through protonated species ([M + H]^+^) (Table 2). Interestingly, the highly related compounds α-, γ-, and β-tocopherols exhibited very different ionization energies (i.e., 80, 140, and 220 V, respectively) (Table 2). 

### 2.3. Method Performance Data

**Figure 1:**
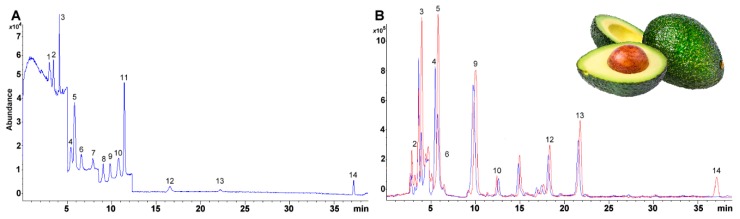
Selected ion monitoring (SIM) chromatogram for (**A**). (**A**) A standard mixture of 14 analytes at 1 μg mL^−1^ for liposoluble vitamins and 0.05 μg mL^−1^ for carotenoids. 1. Retinyl acetate. 2. Ergocalciferol. 3. Cholecalciferol. 4. δ-Tocopherol. 5. γ-Tocopherol. 6. α-Tocopherol. 7. Phylloquinone. 8. Astaxanthin. 9. Lutein. 10. Zeaxanthin. 11. Retinyl palmitate. 12. β-Cryptoxanthin. 13. β-Carotene. 14. Lycopene. (**B**) The saponified avocado sample analyzed using the proposed chromatographic method (blue line), and a sample spiked with 5 μmol for each vitamin and 1 μmol for each carotenoid (red line).

For the vitamin group, the higher sensitivity was exhibited by α-, γ-, and retinyl palmitate (Table 2). On the other hand, carotenoids with a lower limit of detection were astaxanthin, lutein, and zeaxanthin (Table 3). Electrospray analysis of carotenoids, since some years ago, has demonstrated high sensitivity (i.e., in the pmol range) [39]. These compounds, as expressed in the matrix of interest, could be detected as low as 1 μg/100 g dry matter (Table 3). The resolution obtained between the compounds analyzed ranged from 2.18–71.90; the lowest value recommended is 2 (Table 2, Figure 1A) [40]. Several compounds were very close to the theoretical value of 1 (i.e., a perfect peak with a Gaussian distribution and completely symmetrical) though ergocalciferol (1.707), and lutein (1.348) exhibited some tailing. Fronting (leading peak) can also be observed for retinyl acetate (0.709) (Table 3). Column efficiency was very high for compounds eluted above 8 min, such as phylloquinone, zeaxanthin, retinyl palmitate, β-cryptoxanthin, and β-carotene (Table 3). A good baseline definition was observed for all compounds analyzed (i.e., α_s_ ranging from 1.07 to 1.76) (Table 3).

**Figure 2:**
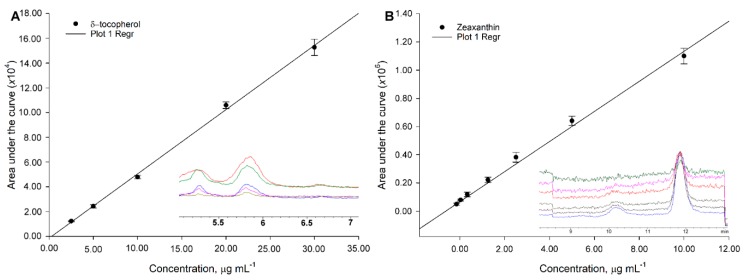
Average calibration curves and error bars depicting variability obtained for two of the target analytes (**A**) δ-tocopherol and (**B**) zeaxanthin. Mean and standard deviations (used as error) calculated from *n* = 3 independently constructed calibration curves injected on different days.

The method was successfully applied to avocado fruits (Figure 2B). The lack of available certified reference materials for matrices such as fruits obliges the use of spike solutions to demonstrate both matrix effects (if any) and extraction efficiency of vitamins and carotenoids in avocado specifically.

### 2.4. Performance during Saponification

Saponification-wise, using the same starting mass and sample, we proceeded to optimize the conditions needed (i.e., base concentration and time) to improve analyte recovery. The differential analysis showed that, overall, samples treated at 80 °C for one hour and using 1 mol KOH L^−1^ exhibited the best results in the case of the analytes of interest, for avocado (Table 4). Variables tested showed a profound and significant effect over analyte recovery (*p* < 0.05 for all cases) (Table 4). Reaction parameters must be optimized to ensure proper hydrolysis within the complex, considerably oily (ranging from 35.3 to 39.1 g fat/100 g dry weight basis), food matrix that is found in the ripe avocado fruit [41]. Carotenoids are increasingly sensitive to heat. Hence, hot saponification was carried out using an organic solvent with a relatively low boiling point [6] (i.e., 78.37 °C at Standard Pressure and Temperature [STP] for ethanol) and pyrogallol was used as a radical sink to protect the compounds of interest. However, the improvement in recovery was achieved by increasing the temperature to 80 °C. A procedure that might be justified as (i) the thermal effect must be sufficient to break the cell walls from the avocado fruit and provide a rapid molecular diffusion to promote reaction; (ii) higher temperatures increase solubility of lipophilic compounds and enhance kinetics of saponification (i.e., favors localized “hot spots” which deliver sufficient energy for the molecules to react) [42]. We chose hot saponification to diminish reaction time; a similar approach has been reported elsewhere [43].

### 2.5. Quantification, Linearity, and Calibration Curve Construction

Five-point calibration curves were constructed to quantitate the analytes. Slopes (*m_x_*) ranging from 4.05 × 10^3^ (cholecalciferol) to 5.51 × 10^4^ (ergocalciferol) and 5.45 × 10^4^ (β-cryptoxanthin) to 6.49 × 10^6^ (zeaxanthin) for vitamins and carotenoids, respectively, were obtained. Determination coefficients spoke toward excellent linearity ranging from 0.9906 to 0.9964 (Figure 2A,B). For vitamins, standard calibration curves were constructed as follows: from 1.00 to 10.00 mg L^−1^ for retinyl acetate, ergocalciferol, γ-, and α-tocopherol and retinyl palmitate; from 2.50 to 30.00 mg L^−1^ for cholecalciferol, δ-tocopherol, and phylloquinone. Finally, considering lower concentrations found in the target matrix and carotenoid sensitivity, standard calibration curves were constructed as follows: 0.05 to 0.80 mg L^−1^ for astaxanthin, β-cryptoxanthin, and β-carotene and from 0.1 to 2 mg L^−1^ for zeaxanthin and lutein (Figure 2A,B). Concentrated stock solutions were prepared by dissolving the solid standard in 2-propanol for vitamins and chloroform for carotenoids; dilutions performed after that were matched with the mobile phase (i.e., the gradient at the start of the chromatographic run).

### 2.6. Analyte Recovery and Method Accuracy

Overall, vitamin recovery, in spiked avocadoes, exhibited better performance than for carotenoids (except for β-carotene with a recovery of 108.63%). Structurally, the lower recoveries (ranging from 43.80% to 63.68%) were found for those carotenoids that were oxygenated (Table 5). Noteworthy, when zeaxanthin and β-cryptoxanthin were extracted using a mixture of ethyl ether, and hexane (50:50) obtained/experimental mass increased between 12.35% and 15.56%. Furthermore, when nitrogen flushing was performed, in conjunction, an additional increment between 13.35% to 19.66%, was observed (data not shown). Carotenoids, among several mechanisms of transformation [44,45], are susceptible to thermal degradation [46,47], β-carotene may suffer from symmetrical oxidative cleavage (which generates, the apocarotenoid, retinol) [48], and β-cryptoxanthin can undergo light-induced oxidation and isomerization [49].

### 2.7. Mass Spectra Analysis

**Figure 3:**
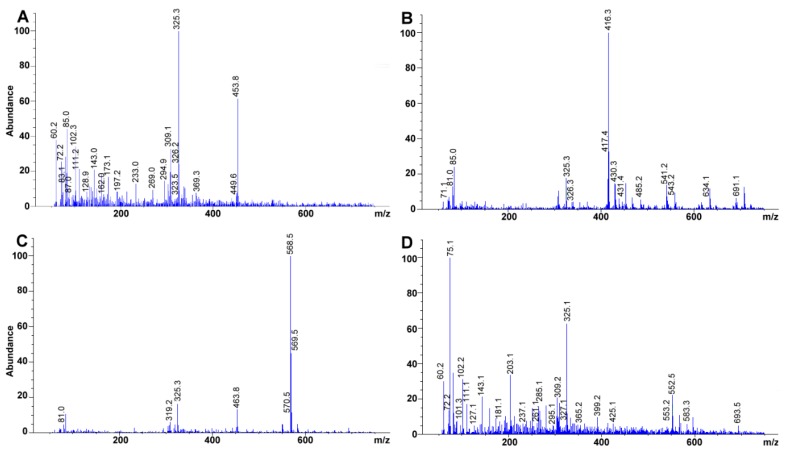
Experimental mass spectra obtained for (**A**) retinyl acetate, (**B**) γ-tocopherol, (**C**) Lutein, (**D**) β-cryptoxanthin. Total ion chromatograms at 100 mg L^−1^ each.

Retinyl acetate and β-cryptoxanthin showed, under our conditions, a higher degree of fragmentation compared to tocopherol and an oxygenated carotenoid. Additional to the signals analyzed above for retinoids, ion 369.3 represented the retinyl acetate molecular ion plus a potassium ion, [M + K]^+^ (Figure 3A). On another hand, tocopherols usually are characterized to present few fragmentation processes [51] (Figure 3B). Major ions are formed by cleavage through the non-aromatic portion of the chromanol ring, both with and without hydrogen transfer and by a loss of the isoprenoid side chain [51]. In contrast, we found none of the usual fragments reported elsewhere for the fragmentation of γ-tocopherol (e.g., C_9_H_11_O_2_^+^ *m*/*z* 151 amu; [51]) probably because the total ion chromatogram was obtained using lower energy than required. Also, there may have been some instrument limitations as a single quadrupole was used throughout the experiments, and the fragmentation ions were the result of molecule degradation in the ion source not in the collision chamber used during tandem mass spectrometry. Hence, the lack of tandem mass detection justified the absence, in some cases, of multiple fragmentation patterns [52]. However, the molecular ion [M+] for γ-tocopherol was unmistakable and could be used to differentiate nuances among tocopherols (e.g., the difference between γ-tocopherol and α-tocopherol is a methyl group in the aromatic ring (Δ15 amu)). As an example of calciferol identification, cholecalciferol total ion chromatogram (TIC)-mass spectra showed relevant fragments at 365.1 (loss of H_2_O), 337.1 (subsequent loss of both –CH_3_ from the isopropyl moiety), 301.2, and 227.0 (loss of –CH_3_ and =CH_2_ or C_17_H_23_^3•^) *m*/*z* (data not shown).

On another hand, Atmospheric-pressure chemical ionization (APCI), Fast atom bombardment (FAB), electron ionization (EI), and ESI all have mass-based techniques used to assess carotenoids [53]. While APCI is a popular approach for the ionization of lipophilic compounds, we selected ESI^+^. In this scenario, some modifications were introduced into the mobile phase to enhance ionization. In this specific case, formic acid was selected as it has been described to decrease signal suppression [54]. For the case of lutein the signal 551 *m*/*z* (i.e., [M + H − 18] which corresponds to the loss of H_2_O) and 463 *m*/*z* ([M + H − 106], loss of two water molecules and xylene from the polyene chain) were observable ([53,55]; Figure 3C). Additionally, as zeaxanthin and lutein differed from each other by a position of a double bond, in a ε-ring, they rendered similar spectra [53]. Further fragmentation for both compounds responded to the loss of said rings. Finally, for β-cryptoxanthin, signal 552.5 ([M+]) and 553.2 *m*/*z* ([M + H]^+^) were evident, fragments 425.1 [C_32_H_40_]^+^ and 399 [M + H − 153]^+^ *m*/*z* responded to the partial breakup of both β-rings and the complete loss of the oxygenated β-ring plus a methyl group (Figure 3D).

### 2.8. Chromatographic Separation of Tocopherol Isomers

**Figure 4:**
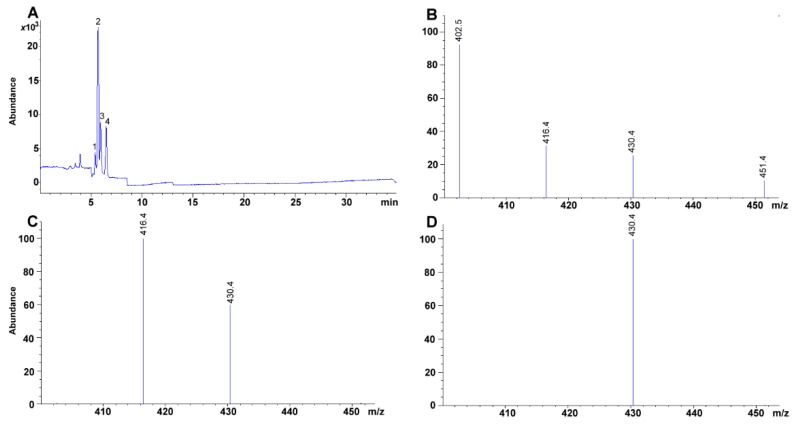
(**A**) Chromatogram of a mixture of tocopherols (W530066 70, 13, 523, 105 mg g^−1^ for α- (peak 4), β- (peak 2), γ- (peak 3), and δ-tocopherol (peak 1), respectively). Selected [M + H]^+^ ion for (**B**) δ-tocopherol (402.1 *m*/*z*), (**C**) β/γ-tocopherol (416.4 *m*/*z*), and (**D**) α-tocopherol (430.4 *m*/*z*). Selected ion monitoring (SIM) at 700, 130, 5230, 1050 μg mL^−1^.

Under our chromatographic conditions, tocopherol isomers were easily segregated (Figure 4A). Using the tocopherol mixture containing α-, β-, δ-, and γ-tocopherols, we were able to assess further that no additional [M+] signal was necessary for the detection of β-tocopherol, as β- and γ-tocopherol share the same molecular mass (Figure 4B,C). The signal for β-tocopherol was observed at a *t_R_* of 5.387 min (Figure 4A). Hence, the four tocopherol isomers eluted as follows: δ-, β-, γ-, and α-tocopherol (Table 1, Figure 4A).

### 2.9. Method Application in Real Samples

Simmonds variety characterizes itself for having an oblong oval to pear-shape large-sized fruit of light green-colored smooth skin, and a seed of medium size, usually tight. Meanwhile, Guatemala has a medium to large size and nearly round shape, smooth, thick, granular, and green skin with a small seed. Finally, Hass fruit possesses an ovoid to pear shape, is of medium size, with tough, leathery, pebbled, thin, dark purple to black (when ripe) skin, with a small seed [30,56].

We found in avocado both non-provitamin A (lutein and zeaxanthin) and provitamin A (β-carotene and β-cryptoxanthin) carotenoids [57] (Table 6). All carotenoids encountered have been previously identified for the fruit (see, for example, [11,20,21,23,28,29]). Our data indicated that, overall, all three varieties of avocado exhibited concentrations, of carotenoids and vitamins, in line (except for provitamin A carotenoids which levels were considerably higher for Costa Rican avocadoes) with those reported elsewhere [28]. Considering the above, Costa Rican avocado would supply to diet retinol equivalents [58] ranging from 1085.21 to 425.01 µg on a dry weight basis. Though three different varieties of avocado have been examined, it should be clear that any number of factors can affect the concentrations of such molecules, including edaphoclimatic conditions, genotypic differences, and nutritional status of the plants [59]. Hass and Guatemala varietals showed a somewhat similar profile for both fat-soluble vitamins as carotenoids. Meanwhile, the Simmonds variety showed higher levels for almost all analytes tested (except for lutein) (Table 6). Interestingly, the δ/α ratio of isomers is close to 1 for all varieties (i.e., 0.75–1.22).

### 2.10. Method Application in Other Samples

**Figure 5:**
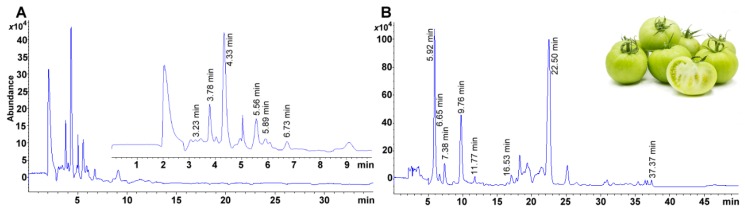
(**A**) Infant formula with certified values for fat-soluble vitamins. The box shows the amplification of the region from 1 to 10 min of the said chromatogram. FAPAS^®®^ reference material TYG009RM. Vitamin A, vitamin D_3_, and vitamin E at 508 ± 12, 7.16 ± 0.33, and 10,900 ± 400 μg/100 g. (**B**) Non-saponified sample of green tomatoes extracted after mechanical shearing and chloroform extraction and analyzed using the proposed method.

As stated before, no standard reference material is available for avocado. However, as a quality control material, we subjected our method to infant formula to further assess method accuracy. We obtained values according to the provider, and no appreciable matrix effects were observed for this food either (Figure 5A). Also, our method showed promise to extrapolate to other matrices, especially fruits. Unambiguous signals of several vitamins of interest can be seen when a chloroform extract obtained from green tomatoes was injected (Figure 5B).

## 3. Materials and Methods

### 3.1. Reagents

*tert*-Butyl methyl ether (99%, catalog 34875, MTBE, chromatographic grade), methanol (≥99.9%, catalog 646377, MeOH, chromatographic grade) and potassium hydroxide (ACS reagent, catalog 1050210250) were acquired from Merck Millipore (Burlington, MA, USA). Retinyl acetate (catalog 46958), retinyl palmitate (catalog 46959-U), cholecalciferol (catalog C9774), ergocalciferol (catalog 47768), 3-phytylmenadione catalog (95271), α-tocopherol (catalog 47783), δ-tocopherol (catalog 47784), γ-tocopherol (catalog T1782), tocopherols (mixed, W530066), lutein (catalog 071068), β-carotene (catalog C4582), β-cryptoxanthin (≥97%, catalog C6368), zeaxanthin (catalog 14681), all-*trans*-astaxanthin (catalog 41659), and lycopene (catalog 75051) from Sigma-Aldrich (unless stated otherwise, all standards were of analytical grade, St. Louis, MO, USA). Chloroform (ACS reagent, catalog 366919), ethanol (200 proof, ACS reagent, ≥99.5%, 459844), 2-propanol (HPLC Plus, 650447), pyrogallol (ACS reagent, ≥99%, catalog 16040), and sodium sulfate (anhydrous, granular, free-flowing, Redi-Dri™, ACS reagent, ≥99%, catalog 798592) were also acquired from Sigma-Aldrich.

### 3.2. Sample Treatment and Preparation

Three different varieties of avocado collected from Costa Rica farms, two varietals from low and one highland, i.e., Simmonds (from 0 to 1000 m amsl), Guatemala (from 600 to 1500 m amsl), and Hass (from 1000 to 2000 m amsl), respectively [30,56]. Three batches of each variety were analyzed, each with eight days of maturation. A previously homogenized subsample (2 g) of freeze-dried material was weighed, and chloroform (20 mL, ACS reagent, Sigma-Aldrich 366919) was added. After that, the mixture was stirred continuously (30 min) using an Ultraturrax^®^ (T25, at 7500 rpm, IKA Works Staufen, Germany). The remnant suspension was centrifuged, and the supernatant liquid recollected. The extraction procedure was repeated twice. The solvent was evaporated using a rotary evaporator (Multivapor™ P-6, Büchi, Flawil, Switzerland) until the lipid fraction was attained, which was then processed immediately for saponification.

### 3.3. Optimization of Saponification Conditions

An additional experiment was performed to enhance the recovery of the analytes of interest during the saponification reaction. The experimental design consisted of maintaining constant reaction time (1 h), the concentration of radical protection agent (0.1 g/100 mL), and the extraction solvent (hexane). Base concentration was contrasted (1 vs. 2 mol KOH L ethanol^−1^ [43] for each temperature), and the temperature was progressively increased (60, 80, and 95 °C). The conditions that rendered the most recoveries were selected to process the samples.

### 3.4. Sample Saponification

A small portion of the fat fraction (0.3 g) was weighed and quantitatively transferred to a conical centrifuge tube (50 mL, CLS430829, polypropylene, Corning^®^, New York, USA). Afterward, KOH in ethanol (1 mmol mL^−1^) and containing pyrogallol (0.1 g/100 g) was added (10 mL). The mixture was let to saponify (during 1 h at 80 °C) in a heat bath (1229U55, Boekel Scientific, Feasterville, PA, USA). The resulting mixture was let to cool to room temperature and transferred to a Squibb separatory funnel (PYREX^®^, 250 mL, Corning^®^ 6402). The above ethanol/aqueous layer was subjected to a liquid-liquid extraction using hexane. The extraction procedure was repeated twice. The totality of the organic solvent layer was then collected and filtered through sodium sulfate (used as a desiccant). The resulting solution was evaporated to dryness under a nitrogen flow (Ultra-High Pure Nitrogen was purchased from Praxair Technology Inc., Danbury, Connecticut, USA) and then reconstituted with MTBE (1 mL) and 2-propanol (1 mL) used at the start of the chromatographic separation and transferred to an HPLC vial (Agilent technologies, Santa Clara, CA, USA).

### 3.5. Stationary Phase and Selection of Chromatographic Conditions

The major obstacle in vitamin separation is the segregation of isomers. With this in mind, as a starter setup, we used a mobile phase based on MeOH and water using an eight carbon-based alkyl stationary phase (0.75 mL min^−1^, Eclipse Plus C_8_, 4.6 mm ID × 150 mm, 3 μm, Agilent Technologies). As the resolution was insufficient, a C_18_ column was selected, and only flow was modified (1 mL min^−1^, Eclipse Plus C_18_, 4.6 mm ID × 150 mm, 3 μm, Agilent Technologies). Then we substituted the column for a C_30_, removed water, and used a less polar solvent in acetonitrile and 2-propanol, reducing yet again the solvent flow (0.5 mL min^−1^) (Table 1). Finally, retaining a similar proportion of MeOH, we substituted acetonitrile and isopropanol for MTBE. The C_30_ column was kept as it already had excellent capabilities reported for highly lipophilic compounds (e.g., carotenoids) [39].

### 3.6. Chromatographic Conditions

All assays performed using an Agilent Technologies LC/MS system equipped with 1260 infinity quaternary pump (61311C), column compartment (G1316A), automatic liquid sampler modules (ALS, G7129A) and a 6120-single quadrupole mass spectrometer with electrospray ionization ion source (Agilent Technologies, Santa Clara, CA, USA). Gradient elution was used to separate all the compounds. The solvent gradient was optimized using MeOH (solvent A) and MTBE (solvent B), both acidified with formic acid (0.1 mL/100 mL). Solvent proportions were set as follows: at 0 min 80% A, at 5 min 80% A, at 7 min 73% A, at 15 min 62.5% A, at 20 min 62.5% A, at 30 min 45% A, at 35 min 10% A, at 40 min 10% A, at 45 min 80% A and 50 min 80% A. Flow rate was kept constant at 0.6 mL min^−1^. Injection volume was held at 10 μL. The column compartment was held at a temperature of 10.0 ± 0.8 °C. Considering the need for the separation of structurally similar compounds, a 30-carbon alkyl chain based chromatographic column was used to achieve the analytical separation (YMC Carotenoid, 4.6 mm ID × 150 mm, S-3 μm, YMC Co., Ltd., Kyoto, Japan).

### 3.7. MS Detection System Conditions

**Figure 6:**
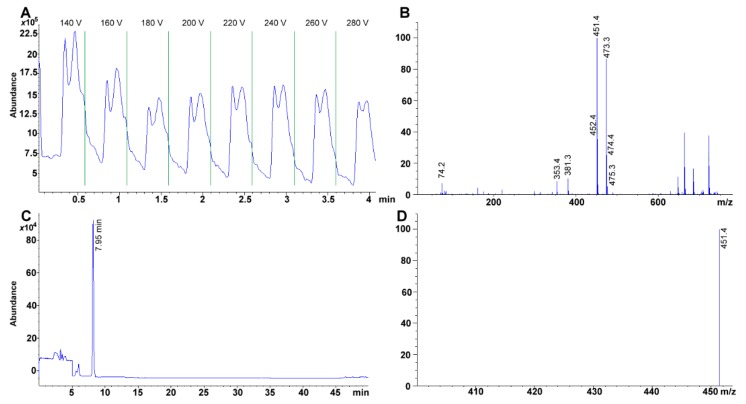
Example of the voltage cycling for phylloquinone to obtain the most sensitivity, data obtained at 100 μg mL^−1^. (**A**) A parameter was selected where the signal delivered the most area under the curve. (**B**) Mass spectra obtained from a total ion chromatogram for phylloquinone at 100 μg mL^−1^. Fragmentation as follows (molar mass 450.7 g mol^−1^): 473.3 ([M + K]^+^), 451.4 ([M + H]^+^), 381.3 ([C_26_H_35_O_2_]^•^), 353.4 ([C_24_H_30_O_2_]^2•^), 225 ([C_15_H_13_O_2_]^•^ and partial alkyl isoprenoid chain [C_16_H_33_]^•^), and 186 (quinone ring, [C_12_H_9_O_2_]^•^) *m*/*z* [60]. (**C**) Chromatogram for phylloquinone was obtained using the selected [M + H]^+^ 451 *m*/*z* and (**D**) selected ion monitoring (SIM) for the target analyte at 20 μg mL^−1^.

The fragmentor was initially cycled to assess the voltage (from 20 to 300 V) that rendered the highest sensitivity for the compounds; omitting column interaction (Figure 6A). Afterward, total ion chromatographs (TIC) allowed us to obtain the MS spectra for each of the compounds (scan mode using a mass range and detector gain set to 50–750 *m*/z, and 10.00, respectively) (Figure 6B,C). Each TIC was used to identify the molecular ion signal. Drying gas, nebulizer pressure, drying gas temperature, and capillary voltage was set, respectively, to 12.0 L min^−1^, 50 psi, 350 °C, 4000 V for positive ion mode electrospray ionization (ESI^+^). Selected ion monitoring was used to corroborate each compound identity, remove interferences and improve sensitivity (SIM mode with peak width and cycle time set to 0.05 min, and 0.30 s cycle^−1^, respectively) (Table 2, Figure 6D).

**Figure 7:**
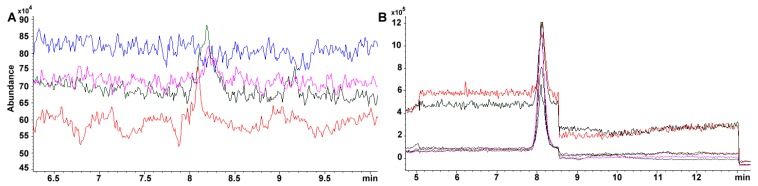
Calibration curve and sensitivity comparison using (**A**). TIC and SIM modes for vitamin K_1_ both signals tested at 137.8, 68.9, 34.4, 17.2, 8.61, and 4.31 mg L^−1^ and using a fragmenter of 140 V. No signal is noticeable at 17.2 mg L^−1^ for TIC (blue line in panel (**A**)). Meanwhile, a calibration curve is easily constructed in SIM mode with the lowest point in 4.31 mg L^−1^ (purple line panel (**B**)).

Sensitivity is greatly improved using a SIM targeted scan. For example, the same standard 34.4 mg phylloquinone L^-1^ in TIC throws 37870 vs. 457785 area under the curve in SIM. Furthermore, within curve sensitivity reaches only 24.1 mg L^−1^ for TIC while the signal for 4.31 mg L^−1^, in SIM, is still appreciable (i.e., 80471 area under the curve, 0.54 mg L^−1^ within curve sensitivity) (Figure 7A,B). Absolute sensitivity to vitamin K increases almost 50 fold (24.1/0.54). For carotenoids, the change is more dramatic as 100 mg L^−1^ standard has to be prepared in TIC for a detectable signal while 0.136 mg L^−1^ is still noticeable (Figure 2B), which represents ca. 750-fold in increased sensitivity.

Retention times and mass spectra were collected by the centroid of the chromatographic peak. Quantitation was carried out by comparing the peak areas found in the samples with those of standard solutions. The identification and quantification of targeted compounds analyzed by LC-ESI^+^-MS were performed using OpenLab Chemstation C.01.07 (Agilent Technologies) for the processing of MS data sets. Confirmation of target analytes was based on the retention time (± 0.2 min as accepted time deviation), measurement of the molecular ion in a specific timeframe (Table 2). 

### 3.8. Statistical Analysis

Calibration curves parameters (i.e., slopes and intercepts), coefficients of determination, limits of detection, and standard errors were computed as a linear fit model using SAS JMP 13 (Marlow, Buckinghamshire, England). An ANOVA with a post-hoc Dunnet test was used to assess differences among treatments during the optimization of the conditions during saponification. Concentrations obtained using the conditions 1 h and 1 mmol KOH mL ethanol^−1^ at 80 °C, were used as the control parameters; the test considered if the data was below the control, with α = 0.05 significance level. The statistical analysis was performed using IBM SPSS^®®^ Statistics 23 (Armonk, NY, USA).

## 4. Conclusions

The proposed method was regarded as a greener option by replacing chlorinated solvents and allowed two nutritionally relevant families of bioactive compounds (i.e., carotenoids and fat-soluble vitamins) to be analyzed together (which is not usually the case) and offered an adequate resolution in the case of tocopherol and calciferol isomers to improve their differential quantification. We obtained an accurate, sensitive, robust, and highly specific multi-analyte method that was successfully applied to avocadoes, a fruit of high economic value, of dietary interest, and a staple of Latin-American cuisine. The use of separation based entirely on organic solvents and the C_30_ column retention capability rendered a versatile method that can facilitate the incorporation of other pigments that have been reported present in avocado fruit (e.g., neoxanthin, trollichrome, chrysanthemaxanthin) [14,21] to further extend its chemical characterization. Saponification was paramount in the recovery of fat-soluble compounds. Therefore, optimized conditions should be assessed for each matrix to be tested. The method may be extended to evaluate fat-soluble vitamins and carotenoids in other matrices. Mass spectrometry was a crucial tool in enabling the discrimination of structurally related compounds (e.g., all three tocopherol isomers could be easily accounted for in avocado).

## Figures and Tables

**Table 1:** Performance of other stationary phases and conditions tested to try to separate calciferol and tocopherol isomers.

Stationary Phase	C_8_		C_18_		C_30_	
Solvent System	95:5 MeOH:H_2_O		95:5 MeOH:H_2_O		90:7:3 MeOH:CH_3_CN:2-propanol	
Flow, mL min^−1^	0.75		1.00		0.50	
Temperature, °C	50		50		35	
Compound	*t_R_*, min	R_s_	*t_R_*, min	R_s_	*t_R_*, min	R_s_
Retinyl acetate	3.50		4.75		5.42	
Ergocalciferol	4.74	0	8.41		8.96	0.78
Cholecalciferol	4.74	0	8.81	1.12	9.27	0.78
δ-tocopherol	4.67	1.47	8.05	1.20	7.42	2.96
γ-tocopherol	5.17	1.47	9.52	2.07	8.28	1.85
α-tocopherol	7.05		15.14		12.19	
Phylloquinone	7.84		19.87		13.17	
Retinyl palmitate	13.09		48.33		35.22	

**Table 2:** Optimized Mass Spectrometry (MS) parameters for the assayed compounds, in order of *m/z*.

Detector Set Time, min	Compound	*t_R_*, min	Selected SIM Ion, *m*/*z*	Fragmentor, V	Dwell Time, ms
From 0 to 5	Retinyl acetate	2.99	269.3 [C_20_H_29_]^•+^/325.2 [C_20_H_29_OH + K]^+^	100	95
	Ergocalciferol	3.59	398.3 [M + H]^+^	220	
	Cholecalciferol	4.02	385.3 [M + H]^+^	160	
From 5 to 8	δ-tocopherol	5.26	402.5 [M+]	220	71
	γ-tocopherol	5.78	416.4 [M+]	140	
	α-tocopherol	6.61	430.4 [M+]	80	
From 8 to 13	Phylloquinone	8.07	451.4 [M + H]^+^	140	56
	Astaxanthin	9.07	597.4 [M + H]^+^	160	
	Lutein	9.97	569.4 [M + H]^+^	140	
	Zeaxanthin	11.49	568.4 [M+]	140	
After 13	Retinyl palmitate	11.19	269.3 [C_20_H_29_]^•+^/563.4 [M + K]^+^	100	95
	β-cryptoxanthin	16.58	552.6 [M+]	120	
	β-carotene	22.51	536.4 [M+]	120	
	Lycopene	37.39	536.1 [M+]	160	

**Table 3:** Method performance parameters obtained during validation.

Sensitivity												
Compound	LoD, μg L^−1^		LoQ, μg L^−1^		LoD, μg/100 g fat		LoQ, μg/100 g fat		LoD, μg/100 g dry matter		LoQ, μg/100 g dry matter	
Retinyl acetate	3.00 × 10^2^		9.20 × 10^2^		1.00 × 10^2^		3.07 × 10^2^		1.50 × 10^1^		4.60 × 10^1^	
Ergocalciferol	1.00 × 10^2^		2.90 × 10^2^		3.30 × 10^1^		9.70 × 10^1^		0.50 × 10^1^		1.50 × 10^1^	
Cholecalciferol	2.70 × 10^2^		8.20 × 10^2^		9.00 × 10^1^		2.73 × 10^2^		1.40 × 10^1^		4.10 × 10^1^	
δ-tocopherol	1.70 × 10^2^		5.10 × 10^2^		5.70 × 10^1^		1.70 × 10^2^		0.90 × 10^1^		2.60 × 10^1^	
γ-tocopherol	1.30 × 10^1^		3.80 × 10^1^		0.40 × 10^1^		1.30 × 10^1^		0.10 × 10^1^		0.20 × 10^1^	
α-tocopherol	0.70 × 10^1^		2.40 × 10^1^		0.20 × 10^1^		0.80 × 10^1^		0.10 × 10^1^		0.10 × 10^1^	
Phylloquinone	4.30 × 10^2^		1.29 × 10^3^		1.43 × 10^2^		4.30 × 10^2^		2.20 × 10^1^		6.50 × 10^1^	
Astaxanthin	2.20 × 10^1^		1.25 × 10^2^		0.70 × 10^1^		4.20 × 10^1^		0.10 × 10^1^		0.60 × 10^1^	
Lutein	1.00 × 10^1^		2.90 × 10^1^		0.30 × 10^1^		1.00 × 10^1^		0.10 × 10^1^		0.10 × 10^1^	
Zeaxanthin	0.90 × 10^1^		2.80 × 10^1^		0.30 × 10^1^		0.90 × 10^1^		0.10 × 10^1^		0.10 × 10^1^	
Retinyl palmitate	2.40 × 10^1^		7.40 × 10^2^		0.80 × 10^1^		2.47 × 10^2^		0.10 × 10^1^		3.70 × 10^1^	
β-cryptoxanthin	3.30 × 10^2^		9.90 × 10^2^		1.10 × 10^2^		3.30 × 10^2^		1.70 × 10^1^		5.00 × 10^1^	
β-carotene	8.80 × 10^2^		2.67 × 10^3^		2.93 × 10^2^		8.90 × 10^2^		4.40 × 10^1^		1.34 × 10^2^	
Lycopene	1.56 × 10^2^		4.71 × 10^2^		5.20 × 10^1^		1.57 × 10^2^		0.80 × 10^1^		2.40 × 10^1^	
Chromatographic Parameters												
Compound	*t_R_*, min	[[], mg L^−1^		Area, ×10^5^	Height, ×10^4^	Peak Width	Symmetry	R_s_	*k*	α		*N*, ×10^4^
Retinyl acetate	3.00	5.00		8.98	12.96	0.12	0.71	3.67	0.35	1.61		1.08
Ergocalciferol	3.48	0.50		5.34	5.12	0.15	1.71	3.54	0.57	1.39		0.92
Cholecalciferol	3.97	1.00		1.76	1.78	0.13	0.91	7.96	0.79	1.71		1.45
δ-tocopherol	5.21	1.00		0.67	0.63	0.18	0.95	2.85	1.35	1.16		1.34
γ-tocopherol	5.71	1.00		9.07	7.65	0.17	0.77	3.52	1.57	1.22		1.87
α-tocopherol	6.49	1.00		21.99	13.27	0.28	0.99	7.20	1.92	1.33		0.88
Phylloquinone	7.89	1.00		0.29	0.32	0.11	0.80	4.75	2.56	1.18		7.77
Astaxanthin	8.94	0.05		0.78	0.40	0.33	0.88	2.18	3.03	1.11		1.19
Lutein	9.68	0.05		4.19	1.95	0.36	1.35	4.59	3.37	1.19		1.17
Zeaxanthin	11.09	1.00		0.66	0.44	0.25	1.06	2.87	3.40	1.07		3.09
Retinyl palmitate	11.75	0.20		5.68	4.52	0.21	1.08	26.91	4.30	1.49		5.03
β-cryptoxanthin	16.40	0.20		0.03	0.04	0.14	0.80	27.10	6.40	1.40		23.14
β-carotene	22.14	1.00		0.38	0.22	0.29	0.76	71.90	8.98	1.76		9.54
Lycopene	37.39	1.00		0.28	0.34	0.14	1.04	-	-	-		118.31

**Table 4:** Optimization of saponification conditions.

Temperature, °C		60		80		95	
Base Concentration, mol KOH L^−1^		1	2	1	2	1	2
Compound	*t_R_*, min	mg/100 g ^a^					
Ergocalciferol	3.59	18.89 (−0.14)	10.98 (−0.50)	22.06	8.38 (−0.62)	1.74 (−0.92)	ND (−1.00)
δ-tocopherol	5.26	9.65 (−0.89)	88.22 (−0.02)	90.06	67.32 (−0.25)	0.24 (−1.00)	88.17 (−0.02)
γ-tocopherol	5.78	0.45 (−0.93)	0.50 (−0.92)	6.14	2.48 (−0.60)	0.11 (−0.98)	9.62 (0.57)
α-tocopherol	6.61	180.96 (−0.32)	102.35 (−0.62)	267.77	90.77 (−0.66)	74.00 (−0.72)	70.63 (−0.74)
Astaxanthin	9.07	18.69 (−0.26)	16.82 (−0.33)	25.14	17.12 (−0.32)	7.64 (−0.70)	14.16 (−0.44)
Lutein	9.97	0.08 (0.00)	0.07 (−0.13)	0.08	0.12 (0.50)	0.02 (−0.75)	0.12 (0.50)
Zeaxanthin	11.49	26.60 (−0.13)	37.70 (0.23)	30.73	47.44 (0.54)	7.85 (−0.74)	20.38 (−0.34)
β-cryptoxanthin	16.58	24.66 (−0.22)	12.13 (−0.61)	31.50	27.02 (−0.14)	5.54 (−0.82)	16.93 (−0.46)
β-carotene	22.51	18.89 (−0.14)	10.98 (−0.50)	22.06	8.38 (−0.62)	1.74 (−0.92)	ND (−1.00)
*p* values		0.044	0.040	-	0.037	0.011	0.028

^a^ Brackets indicate bias, expressed as a fraction, with respect to the saponification treatment of 1 h at 80 °C using a 1 mol L^−1^ base concentration. ND: not detected.

**Table 5:** Spiked avocado samples and recovery for representatives for fat-soluble vitamins and carotenoids.

Compound	*t_R_*, min	Concentration, µmol		Recovery, % ^a^
		Theoretical/Added	Experimental/Obtained	
Ergocalciferol	3.59	5.00 × 10^1^	4.00 × 10^1^	81.21 (70–110)
α-tocopherol	6.61	4.60 × 10^1^	3.70 × 10^1^	80.43 (70–110)
Phylloquinone	8.07	3.50 × 10^1^	4.20 × 10^1^	117.02 (70–110)
Astaxanthin	9.07	1.70 × 10^1^	1.00 × 10^1^	62.27 (60–120)
Lutein	9.97	0.35 × 10^1^	0.23 × 10^1^	63.68 (60–120)
Zeaxanthin	11.49	0.23 × 10^1^	0.10 × 10^1^	43.80 (60–120)
β-cryptoxanthin	16.58	1.09 × 10^2^	6.50 × 10^1^	59.51 (70–110)
β-carotene	22.51	1.23 × 10^1^	1.33 × 10^1^	108.63 (60–120)

^a^ Brackets represent recovery values recommended by AOAC [50].

**Table 6:** Fat-soluble vitamins and carotenoids obtained from Costa Rican avocadoes.

Compound	Simmonds	Hass	Guatemala
	Concentration, mg/100 g dry Weight Basis		
Ergocalciferol	1.84 ± 0.18	0.31 ± 0.16	0.53 ± 0.33
Cholecalciferol	119.50 ± 1.20	103.50 ± 15.50	108.50 ± 33.40
δ-tocopherol	42.48 ±7.96	8.31 ±1.63	6.16 ± 2.88
γ-tocopherol	4.81 ± 0.48	2.42 ± 0.37	0.92 ± 0.16
α-tocopherol	34.80 ± 14.50	8.16 ± 0.97	8.20 ± 3.67
Astaxanthin	2.23 ± 0.14	0.64 ± 0.23	0.98 ± 0.55
Lutein	6.41 ± 2.03	15.13 ± 8.66	10.79 ± 2.77
Zeaxanthin	0.03 ± 0.01	0.02 ± 0.01	0.02 ± 0.01
β-cryptoxanthin	6.10 ± 1.39	3.37 ± 0.20	1.66 ± 0.85
β-carotene	3.43 ± 2.09	2.28 ± 1.56	1.71 ± 0.21
